# Signalling pathways underlying pulsed electromagnetic fields in bone repair

**DOI:** 10.3389/fbioe.2024.1333566

**Published:** 2024-01-24

**Authors:** Aoao Wang, Xinbo Ma, Jiaqi Bian, Zhenrui Jiao, Qiuyi Zhu, Peng Wang, Yantao Zhao

**Affiliations:** ^1^ Medical School of Chinese PLA, Beijing, China; ^2^ Department of Chemistry, Capital Normal University, Beijing, China; ^3^ Senior Department of Orthopaedics, The Fourth Medical Center of PLA General Hospital, Beijing, China; ^4^ Department of Neurosurgery, The First Medical Center of Chinese PLA General Hospital, Beijing, China

**Keywords:** pulsed electromagnetic fields, osteoblast, osteoclast, mechanisms of osteogenesis, signalling pathway, bone regeneration

## Abstract

Pulsed electromagnetic field (PEMF) stimulation is a prospective non-invasive and safe physical therapy strategy for accelerating bone repair. PEMFs can activate signalling pathways, modulate ion channels, and regulate the expression of bone-related genes to enhance osteoblast activity and promote the regeneration of neural and vascular tissues, thereby accelerating bone formation during bone repair. Although their mechanisms of action remain unclear, recent studies provide ample evidence of the effects of PEMF on bone repair. In this review, we present the progress of research exploring the effects of PEMF on bone repair and systematically elucidate the mechanisms involved in PEMF-induced bone repair. Additionally, the potential clinical significance of PEMF therapy in fracture healing is underscored. Thus, this review seeks to provide a sufficient theoretical basis for the application of PEMFs in bone repair.

## 1 Introduction

Non-union or delayed bone healing is a general orthopaedic disease with difficult healing. The probability of a delayed healing fracture is 5%–10% worldwide (Valiya Kambrath et al., 2020). Multiple bone defects, such as severe injuries, surgical removal of infected bones or tumours, and congenital skeletal anomalies, can reduce the regenerative capacity of bones, thereby affecting patient health and quality of life (El-Rashidy et al., 2017; Collon et al., 2021; Li et al., 2021). Major treatments for bone defects include autologous and allogeneic bone grafting, bone grafting with vascularised tips, the Masquelet technique, and bone tissue engineering (Wang and Yeung, 2017; Hofmann et al., 2020; Zhang et al., 2020; Dalisson et al., 2021). Autologous bone grafting is considered the gold standard for the clinical treatment of bone defects. However, autografting techniques have some unavoidable disadvantages, including limited bone volume, increased bleeding and surgery time, and pain in the donor area (Yu et al., 2020; Zhu et al., 2021; He et al., 2022). Pulsed electromagnetic fields (PEMFs) are non-invasive, safe, and have wide indications. Therefore, they have been increasingly used to treat bone diseases, such as fractures and delayed bone healing, in recent years. This review briefly discusses recent research progress on the application of PEMFs in bone repair, with a particular focus on the molecular mechanisms underlying PEMF-induced bone repair. Furthermore, this review aims to provide a sufficient theoretical basis for the clinical application of PEMFs in bone repair.

## 2 Research progress on PEMF applications in bone repair

Norton et al. (1977) first attempted the electrical stimulation method to treat bone non-union in 1812, achieving some progressive results. However, he did not have the relevant theoretical foundation for the clinical application of this method. In 1953, Yasuda (1977a) reported the presence of piezoelectric effects in bone tissue, leading to a considerably increased focus on electrical signals in bone. Yasuda (1977b) later examined rabbit bone repair using external electrodes, revealing that bone connected to the negative electrode would grow a bone scab in the direction of the anode under electrical stimulation. Furthermore, electrical stimulation can promote bone scab growth without relying on mechanical external forces. These findings led to a preliminary understanding of the role of electrical stimulation in bone growth, repair, and reconstruction. Thus, Qiu et al. (2020) pioneered the use of PEMFs to treat bone fractures in the 1970s, demonstrating that PEMFs promote fracture healing. In 1979, PEMFs were approved by the United States Food and Drug Administration Agency as a safe and effective treatment for non-healing bone (Cadossi et al., 2020).

**FIGURE 1:**
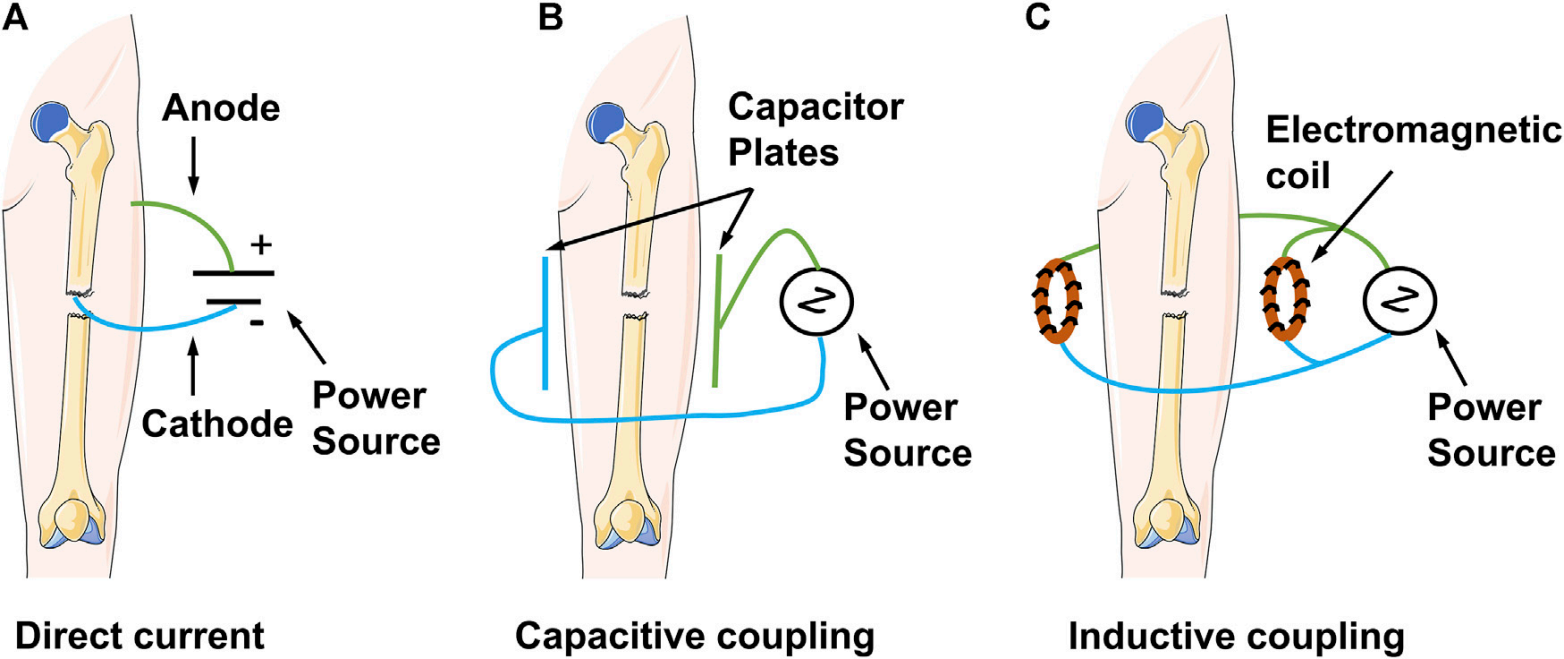
Schematic diagram demonstrating the mechanisms of clinical electrical stimulation devices.

PEMFs play an unusual role in the treatment of fractures, bone defects, and bone non-union. They artificially provide electromagnetic signals to the target area, thereby mobilising the tissues or organs at the site of injury to actively play an osteogenic role. All electromagnetic field devices work by generating a small amount of current within the bone; they only differ in the modes of action. The earliest type of electrical stimulation was invasive direct current (DC) stimulation. This technique utilised a current generator that delivered DC stimulation to a designated area via a metal wire and electrodes. The negative electrode was implanted in the area of bone repair, whereas the positive electrode was placed in the nearby soft tissue (Kooistra et al., 2009). However, the current generator is usually surgically removed after 6–9 months or after healing occurs. Therefore, the wires and electrodes may not be removed; this can lead to complications, such as re-infection and injury (Leppik et al., 2020). Contrastingly, non-invasive capacitive coupling involves placing two capacitive plates on the skin on either side of the fracture area. An external power source is subsequently connected to create an electric field with a voltage gradient between the two plates. Despite the obvious advantages of capacitively coupled stimulation, including small size, light weight, non-invasiveness, and ease of use, patients must change the batteries daily; this can present a problem of patient noncompliance (Cook et al., 2015). Inductive coupling is the basic principle for applying PEMFs. It involves the use of two electromagnetic coils connected to a signal generator over the skin. The coils generate an electromagnetic field that induces a time-varying secondary electric field within the bone to trigger enhanced growth and remodel biological effects on the bone (Yuan et al., 2018) (Figure 1). As a non-invasive physical factor therapy, PEMFs reduce pain, improve bone quality, and improve the functional prognosis of patients. As such, they are recommended as an effective physical therapy factor by domestic and international guidelines (Khalifeh et al., 2018). PEMFs improve bone metabolism by generating a specific frequency and size of pulsed current and using the resonance effect to change the bioelectricity and biomagnetic field of the body. Corresponding biological changes can occur when the frequency of the pulsed current generated by PEMFs matches the cyclotron resonance frequency of key ions (e.g., Na^+^, K^+^, and Ca^2+^), owing to the alteration of ion channel activity (Tong et al., 2017). In addition, high-frequency electric fields can penetrate the cytoplasm to affect mitochondrial activity and regulate energy metabolism levels (Chalidis et al., 2011). Pettersen et al. (2021) recently found that PEMFs markedly contribute to improved osteoblast survival and soluble collagen production. Furthermore, there was no significant difference in pH between stimulated experimental groups and the controls. Similarly, Kim et al. (2006) demonstrated that PEMFs induce osteoblast proliferation and vascular endothelial growth factor (VEGF) production. *In vivo* and *ex vivo* studies have shown the effects of electromagnetic fields on bone density, bone tissue morphology, bone marrow mesenchymal stem cells, osteoblasts, and osteoclasts (Zhou et al., 2019). Table 1 lists recent *in vivo* studies conducted on the use of electromagnetic fields to treat fractures in animal models. Table 2 lists the bioeffects of electromagnetic fields on osteoblasts and osteoclasts *in vitro*. The optimal waveform and parameter regimen for a particular site of fracture, as well as the electromagnetic field sensation and signalling mechanisms in osteoblasts, remain unclear. Therefore, further exploration of these parameters and mechanisms is required to guide clinical treatment (Maziarz et al., 2016; Yan et al., 2022).

**TABLE 1:** Animal-level studies of electromagnetic fields for the treatment of fractures.

Fracture model	Treatment parameters	Results	References	Year
Rabbit Femur	Type: PEMF	-	Smith and Nagel (1983)	1983
	Settings: repetitive pulse-72 Hz			
	Duration: 12 h/day			
Sheep Tibia	Type: PEMF	-	Law et al. (1985)	1985
	Settings: 1.6 mT			
	Duration: 24 h/day			
Horse Tibia	Type: PEMF	+	Kold et al. (1987)	1987
	Settings: asymmetric pulse burst of 30 m duration repeated at 1.5 Hz			
Horse Metatarsal	Type: PEMF	-	Sanders-Shamis et al. (1989)	1989
	Settings: 20 G; 15 Hz			
	Duration: 8 h/day			
Rat Tibia	Type: PEMF	-	Muhsin et al. (1991)	1991
	Duration: 8 weeks			
Horse Metacarpus	Type: PEMF	+	Canè et al. (1991)	1991
	Settings: 28 G; 75 Hz			
Rat Mandible	Type: PEMF	+	Takano-Yamamoto et al. (1992)	1992
	Settings: 1.5–1.8 G; 100 Hz			
Dog Mandible	Type: PEMF	+	Ortman et al. (1992)	1992
	Duration: 1 h/day			
Rat Tibia	Type: PEMF	+	Sarker et al. (1993)	1993
	Duration: 1 h/day			
Rat Spine	Type: PEMF	+	Guizzardi et al. (1994)	1994
	Duration: 18 h/day			
Dog Lumbar spine	Type: PEMF	-	Kahanovitz et al. (1994)	1994
	Settings: 1 G; 1.5 Hz			
	Duration: 0.5–1 h/day			
Rabbit Humerus	Type: PEMF	+	Yonemori et al. (1996)	1996
	Settings: 2 G, 25 µs pulses at 10 Hz			
	Duration: 12 h/day for 14 days			
Rabbits Femur	Type: PEMF	+	Matsumoto et al. (2000)	2000
	Settings: 0.8 mT			
	Duration: 4 h/day			
Rabbits Tibia	Type: PEMF	+	Fredericks et al. (2000)	2000
	Duration: 1 h/day			
Dog Tibia	Type: PEMF	+	Inoue et al. (2002)	2002
	Settings: 0–2.4 G			
	Duration: 4 h/day			
Rabbits Tibia	Type: PEMF	+	Ottani et al. (2002)	2002
	Settings: 8 mT; 50 Hz			
	Duration: 0.5 h/day			
Rabbits Tibia	Type: PEMF	-	Buzzá et al. (2003)	2003
	Settings: pulse width 85 µs			
	Duration: 30 min/day			
Rabbits Tibia	Type: PEMF	+	Fredericks et al. (2003)	2003
	Settings: time-varying field 1.5 Hz			
	Duration: 1 h/day			
Rabbits Tibia	Type: PEMF	+	Shimizu et al. (2004)	2004
	Settings: 1.8 G; 1.5 Hz			
Rat Tibia	Type: PEMF	+	Lirani-Galvão et al. (2006)	2006
	Settings: 30 mW/cm2; 1.5 MHz			
Rabbits Tibia	Type: PEMF	-	Taylor et al. (2006)	2006
	Duration: 1 h/day for 20 days			
Sheep Femur	Type: PEMF	+	Benazzo et al. (2008)	2008
	Settings: 1.5 mT; 75 Hz			
	Duration: 6 h/day			
Rat Tibia	Type: PEMF	+	Grana et al. (2008)	2008
	Settings: 72 mT; 30 Hz			
	Duration: 1 h/day			
Rat Femur	Type: PEMF	+	Puricelli et al. (2009)	2009
	Settings: 41 G			
Rat Tibia	Type: PEMF	+	Shen and Zhao (2010)	2010
	Settings: 8 G; 15 Hz			
	Duration: 2 h/day			
Rabbits Femur	Type: PEMF	+	Aydin and Bezer (2011)	2011
	Settings: 220–260 G			
Rabbit Tibia	Type: PEMF	-	Taylor et al. (2012)	2012
	Settings: asymmetric pulse 1.5 Hz			
	Duration: 20 days continuous			
Rat Tibia	Type: PEMF	-	van der Jagt et al. (2012)	2012
	Settings: 1 G; 5 m pulse; 15 Hz			
	Duration: 2 h/day			
Rat Femur	Type: PEMF	+	Atalay et al. (2015)	2015
	Settings: 1.5 mT; 50 Hz			
	Duration: 6 h/day for 30 days			
Rat Femur	Type: PEMF	+	Oltean-Dan et al. (2019)	2019
	Settings: 6.65 mT; 27.12 MHz			
	Duration: 10 min/day for 14 days			
Rabbit Tibia	Type: PEMF	+	Fredericks et al. (2019)	2019
	Settings: 6.2 mT; 15 Hz			
	Duration: 6 h/day			
Rat Femur	Type: PEMF	+	Umiatin et al. (2021)	2021
	Settings: 1.6 mT; 50 Hz			
	Duration: 4 h/day for 28 days			

**TABLE 2:** Studies of electromagnetic fields bioeffects on osteoblasts and osteoclasts.

Cellular source	Treatment parameters	Results	References	Year
Human Osteoblasts	Type: SEMF	Enhanced mRNA expression of COL1	Heermeier et al. (1998)	1998
	Settings: 6 mT; 20 Hz			
Rat Osteoclasts	Type: PEMF	Inhibition of osteoclastogenesis	Chang et al. (2004a)	2004
	Settings: 7.5 Hz			
	Duration: 0.5, 1, 2 and 8 h/day			
Rat Osteoclasts	Type: PEMF	Increase in osteoclastogenesis	Chang et al. (2005)	2005
	Settings: 7.5 Hz			
	Duration: 0.5, 2, 8 h/day			
Mouse Osteoclasts	Type: PEMF	Increase in cell apoptotic rate	Chang et al. (2006)	2006
	Settings: 7.5 Hz			
	Duration: 8 and 16 h			
Rat Osteoblasts	Type: PEMF	Inhibition of cell proliferation and enhancement of ALP activity	Tsai et al. (2007)	2007
	Settings: 0.32 mT; 7.5 Hz			
Mouse Osteoblasts	Type: SMF	Inhibition of proliferation rate, enhancement of ALP activity	Chiu et al. (2007)	2007
	Settings: 0.1, 0.25, and 0.4 mT			
	Duration: 24 h			
Mouse Osteoblasts	Type: ELF-EMF	Increase in collagen synthesis	Soda et al. (2008)	2008
	Settings: 3 mT; 60 Hz			
Rat Osteoblasts	Type: PEMF	Increase in cell proliferation rate, increase in ALP activity, decrease of percentage of S and G (2)M phase	Wei et al. (2008)	2008
	Settings: 1.55 mT; 48 Hz			
	Duration: 48 h			
Human Osteoblasts	Type: SMF	Inhibition of ALP activity	Denaro et al. (2008)	2008
	Settings: 0.9 μT			
	Duration: 3, 7, and 14 days			
Rat Osteoblasts	Type: PEMF	Induce the uptake of intracellular calcium	Zhang et al. (2010)	2010
	Settings: 0.8 mT; 50 Hz			
	Duration: 9 min			
Human Osteoblasts	Type: SMF	Inhibition of cell proliferation rate, increase in ALP activity	Yang et al. (2010)	2010
	Settings: 400 mT			
	Duration: 72 h			
Mouse Osteoblasts	Type: PEMF	Release more NO, enhancement of cell proliferation, inhibition of ALP activity	Lin and Lin (2011)	2011
	Settings: 1.5 mT; 75 Hz			
	Duration: 9 h			
Rat Osteoblasts	Type: SEMF	Inhibits osteoblast proliferation and promotes osteoclast differentiation and mineralization	Zhou et al. (2011)	2011
	Settings: 0.9–4.8 mT; 50 Hz			
	Duration: 30 min/day for 15 days			
Mouse Osteoblasts	Type: PEMF	Increase in cellular proliferation, positive effects on differentiation	Esmail et al. (2012)	2012
	Settings: 4 mT; 15 Hz			
	Duration: 30 min/day for 2 days			
Human Osteoclasts	Type: PEMF	Less differentiated phenotype, inhibition of TRAP activity	Barnaba et al. (2012)	2012
	Settings: 0.4 mT; 50 Hz			
	Duration: 7 days			
Human Osteoblasts	Type: PEMF	Enhanced cell proliferation and increased ALP activity	Barnaba et al. (2013)	2013
	Settings: 0.4 Mt; 14.9 Hz			
	Duration: 72 h, 7 and 10 days			
Rat Osteoblasts	Type: SEMF	Inhibition of proliferation rate at day 3, increase in osteogenic differentiation at day 9 and 12	Zhou et al. (2014)	2014
	Settings: 1.8 mT; 50 Hz			
	Duration: 30 min/day			
Mouse Osteoclasts	Type: SEMF	Increase in osteoclastogenesis	Hong et al. (2014)	2014
	Settings: 1 mT; 7.5 Hz			
	Duration: 4 and 5 days			
Mouse Osteoclasts	Type: PEMF	Inhibition of the number of osteoclast-like cells	He et al. (2015)	2015
	Settings: 3.8 mT; 8 Hz			
	Duration: 40 min/day for 3 days			
Human Osteoblasts	Type: PEMF	Increase osteoblast viability and maturation	Ehnert et al. (2015)	2015
	Settings: 10–90.6 Hz			
	Duration: 3 times/week for 21 days			
Human Osteoblasts	Type: PEMF	Enhanced expression of OCN, ALP and RUNX-2	Hiemer et al. (2016)	2016
	Settings: 3 mT; 20 Hz			
	Duration: 135 min/day for 3 days			
Mouse Osteoblasts	Type: SEMF	Enhancement of immature osteoblasts proliferation at days 1, 5, and 10 increase ALP expression at days 5 and 14	Bique et al. (2016)	2016
	Settings: 1 mT; 50 Hz			
	Duration: 30 min/day			
Human Osteoblasts	Type: SMF	Enhancement of osteoblastic differentiation	Kim et al. (2017)	2017
	Settings: 15 mT			
	Duration: 3, 7, and 14 days			
Mouse Osteoclasts	Type: PEMF	Inhibition of osteoclast formation and maturation	Wang et al. (2017)	2017
	Settings: 0.5 mT; 15 Hz			
	Duration: 2 h/day for 7 days			
Mouse Osteoblasts	Type: SMF	Increase in osteoblast differentiation and mineralization	Yang et al. (2018)	2018
	Settings: 500 nT and 0.2 T			
	Duration: 8 days			
Human Osteoclasts	Type: PEMF	Inhibition of osteoclastogenesis	He et al. (2018)	2018
	Settings: 15 Hz			
	Duration: 4 h/day for 8 days			
Rat Osteoblasts	Type: PEMF	ALP activity is elevated and the area of alizarin staining is increased	Shao et al. (2019)	2019
	Settings: 0.6 mT; 50 Hz			
	Duration: 1.5h/day			
Rat Osteoblasts	Type: PEMF	PEMF caused a specific high expression of AP-1 in irradiated osteoblasts	Yan et al. (2022)	2022
	Settings: 2 mT; 15 Hz			
	Duration: 2 h/day			

SEMF: sinusoidal electromagnetic field; ELF-EMF: extremely low frequency-electromagnetic field; SMF: static magnetic field; COL1: type I collagen; ALP: alkaline phosphatase; TRAP: tartrate-resistant acid phosphatase; OCN: osteocalcin; RUNX-2: Runt-related transcription factor 2.

## 3 Mechanisms underlying osteogenesis induced by PEMFs

PEMFs primarily function through electromagnetic signals that can activate cell membrane ion channels and regulate cell signalling pathways to promote the directional migration and differentiation of osteoblasts, nerve regeneration, and blood vessel growth. Furthermore, they promote bone repair, a considerably complex process. However, their specific mechanisms remain unclear. At a macroscopic level, the introduction of electromagnetic fields creates an energetic electric field in the body to regulate cell proliferation and differentiation, thereby mediating bone repair (Zhao et al., 2006; Isaacson and Bloebaum, 2010; Reid and Zhao, 2014; Vadlamani et al., 2019). At a microscopic level, electromagnetic signals may affect cell membrane polarisation or ionic displacement, thereby altering intracellular homeostasis and regulating some cellular behaviours (Sundelacruz et al., 2008). PEMFs induce faster passage of ions through the cell membrane, contributing to signalling in the interior of the cell and regulating membrane potential and the cytokinesis axis for osteogenesis (Martin-Granados and McCaig, 2014; Kim et al., 2020). These findings demonstrate that electromagnetic fields play an important role in bone reconstruction. Thus, studying the mechanism of electromagnetic fields affecting bone regeneration and how they affect the behaviour of bone cells and regulate cell physiological activities could facilitate an in-depth understanding of the bone repair process.

### 3.1 Effects of PEMFs on bone cells

The positive osteogenic effects of PEMFs at the cellular level demonstrated its potential mechanism in promoting osteogenesis (Huang et al., 2008; Leppik et al., 2020; deVet et al., 2021). The use of capacitively coupled electric fields on human cranial osteoblasts revealed substantial upregulation of the expression of many transforming growth factor-β (TGF-β) family genes (TGF-β1, β2, and β3) and fibroblast growth factor-2 (FGF-2) and enhanced ALP mRNA expression. The proteins encoded by these genes play pivotal roles in fracture healing. Bodamyali et al. (1998) used PEMFs to verify alterations in mRNA levels of TGF-β and bone morphogenetic protein 2 (BMP-2) in osteoblasts. The effects of varying pulse waveforms of PEMFs on osteoblast proliferation and differentiation showed variability. Zhou et al. observed that square electromagnetic fields promoted osteoblast proliferation but did not support osteogenic differentiation. Conversely, SEMFs inhibited cell proliferation while enhancing osteogenic differentiation. In contrast, triangular electromagnetic fields had no effect on cell proliferation but induced the strongest osteogenic activity (Zhou et al., 2014). Moreover, PEMFs can modulate osteoclast formation, differentiation, and activity by altering the electromagnetic frequency. Hong et al. (2014) discovered that 45 Hz PEMFs inhibited RANKL-induced IκB phosphorylation to hinder osteoclast formation. Conversely, 7.5 Hz PEMFs promoted osteoclast differentiation by activating extracellular regulated protein kinase (ERK) and p38 mitogen-activated protein kinase (MAPK). Although scholars are increasingly investigating the effects of PEMFs on cells, the precise mechanisms underlying cellular sensitivity, interpretation, and transformation of electromagnetic signals necessitate further investigation (Eischen-Loges et al., 2018). Skeletal and bone tissue encompass numerous cell types, including mesenchymal stem cells, chondrocytes, chondroblasts, osteoblasts, and osteoclasts. Electromagnetic fields influence bone and bone tissue by modulating the behaviour of these bone-related cells. Given the significant roles of osteoblasts and osteoclasts in fracture healing and the maintenance of bone homeostasis, this review comprehensively evaluates the effects and potential mechanisms of PEMFs on osteoblasts and osteoclasts.

#### 3.1.1 Osteoblasts

**FIGURE 2:**
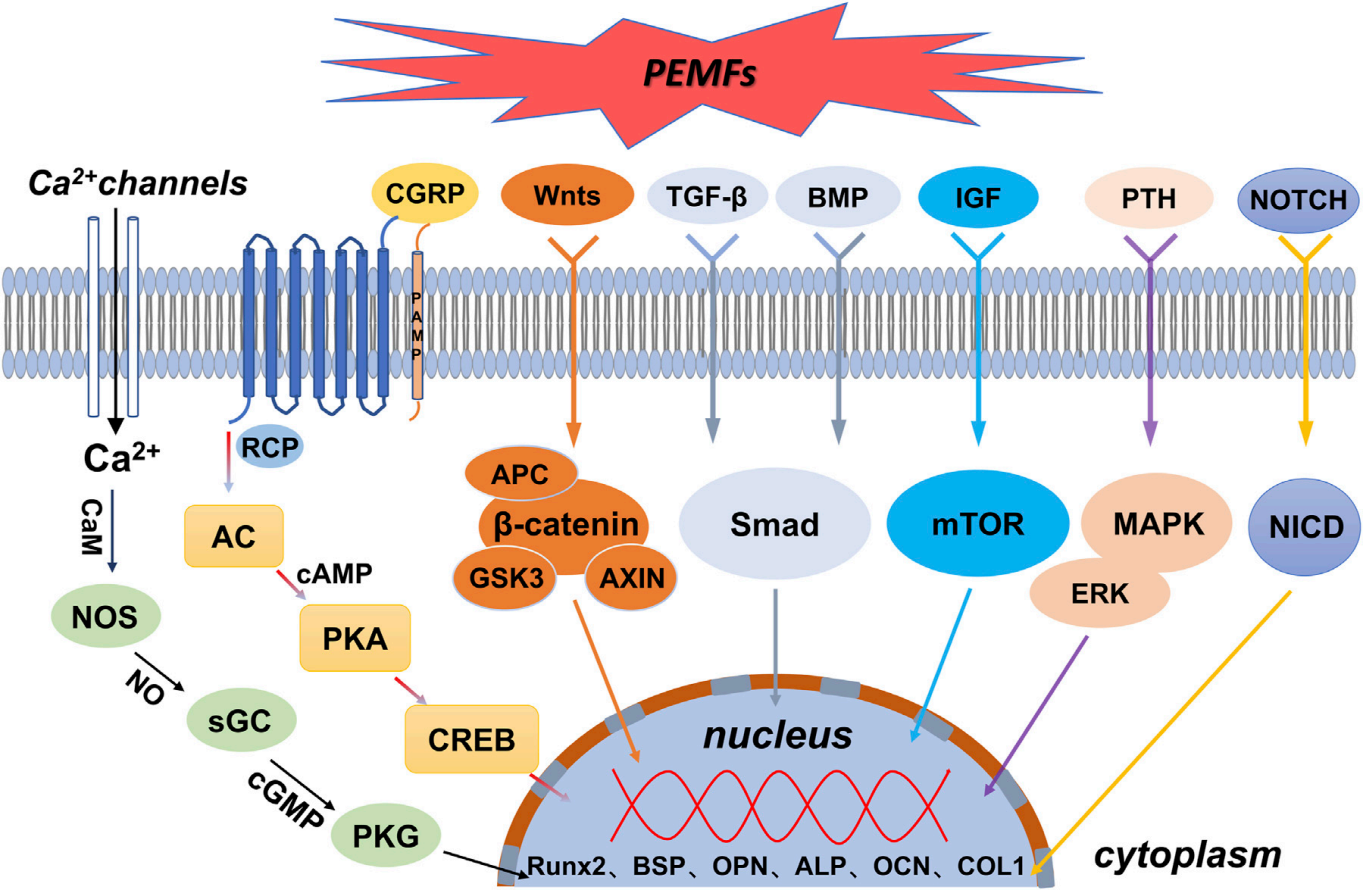
Schematic representation of molecular pathways activated by PEMFs in osteoblasts. AC = adenylyl cyclase. ALP = alkaline phosphatase. APC = adenomatous polyposis coli. BMP = bone morphogenetic protein. BSP = bone sialoprotein. AXIN = axis inhibition protein. CaM = calmodulin. cAMP = cyclic adenosine monophosphate. cGMP = cyclic guanosine monophosphate. CGRP = calcitonin gene-related peptide. COL1 = type I collagen. CREB = cyclic adenosine monophosphate-responsive element binding protein. ERK = extracellular regulated protein kinases. GSK3 = glycogen synthase kinase 3. IGF = insulin-like growth factor. MAPK = mitogen-activated protein kinase. mTOR = mammalian/mechanistic target of rapamycin. NICD = notch intracellular domain. NOS = nitric oxide synthase. OCN = osteocalcin. OPN = osteopontin. PKA = protein kinase A. PKG = protein kinase G. PTH = parathyroid hormone. Runx2 = runt-related transcription factor 2. sGC = guanylyl cyclase. Smad = *drosophila* mothers against decapentaplegic. TGF-β = transforming growth factor-β.

Osteoblasts are direct contributors to bone formation that also regulate the proliferation and differentiation of osteoclasts through various mechanisms. This pivotal role makes these cells essential for bone regeneration. Nonetheless, the impact of PEMFs on osteoblasts remains a subject of contention. PEMFs exhibit a ‘window effect’ and can yield reproducible osteogenic outcomes. In addition, different intensities of PEMFs and varying time points chosen for analysis may have different effects. However, most studies have shown that PEMFs can promote the expression of genes and proteins through specific signalling pathways in osteoblasts, thereby accelerating their proliferation (Chang et al., 2004b; Ercan and Webster, 2008; Lin and Lin, 2011), differentiation (Eischen-Loges et al., 2018; Hou et al., 2019; Leppik et al., 2019), and mineralisation (Wiesmann et al., 2001; Qi et al., 2018). Figure 2 shows a schematic representation of molecular pathways activated by PEMFs in osteoblasts.

Electromagnetic fields regulate the expression of downstream osteogenesis-related genes and proteins by activating the Wnt/β-catenin signalling pathway, thereby enhancing the functions of associated osteoblasts, such as proliferation, differentiation, and mineralisation, to promote osteogenesis (Zhai et al., 2016). Mounting evidence indicates a close association between PEMFs and the Wnt/β-catenin signalling pathway in osteogenesis. For instance, both gene and protein expression of the classical Wnt/β-catenin signalling pathway (including Wnt1, LRP6, and β-catenin) were significantly upregulated following exposure to PEMFs during the proliferation and differentiation stages of MC3T3-E1 cells (Zhou et al., 2015; Jing et al., 2016). Moreover, PEMF intervention reduced the expression of dickkopf1 (DKK1), which typically inhibits the Wnt signalling pathway (Fathi and Farahzadi, 2017). In addition, PEMF-enhanced Wnt/β-protein signalling considerably increased the expression of proliferation-phase-related target genes *CCND1* and *CCNE1* and differentiation-phase-related genes *ALP*, *OCN*, *COL 1*, and *RUNX*-*2*. These genes accelerated osteoblast proliferation, differentiation, and mineralisation (Sun et al., 2010; Clark et al., 2014; Zhai et al., 2016; Fathi and Farahzadi, 2017).

Ion channels are transmembrane proteins embedded in the lipid bilayer of the cell membrane. Furthermore, they are hydrophilic micropore channels that allow selective passage of ions through the cell membrane. Ca^2+^ is an important cellular mediator with roles in several activities, such as cell proliferation, differentiation, and apoptosis. The transient increase in intracellular Ca^2+^ is an immediate effect of electrical signal stimulation on cellular response. Various electrical stimuli (e.g., DC, PEMFs, and piezoelectric stimulation) can alter intracellular Ca^2+^ levels by inducing Ca^2+^ influx or release from intracellular stores. This promotes osteoblast proliferation and osteogenic protein expression, potentially due to the accumulation of charge on the cell membrane, ultimately leading to the opening of voltage-gated calcium channels (VGCCs) (Kim et al., 2009; Xu et al., 2009; Shuai et al., 2018; Cai et al., 2021). Exposure to electromagnetic fields directly activates VGCCs in the plasma membrane, and the channel can trigger multiple regulatory responses through the enzymatic action of Ca^2+^/calmodulin (CaM)-dependent nitric oxide synthase (NOS) (Pall, 2013). Xu et al. used signalling pathway inhibitors to conclude that capacitively coupled electrical stimulation, which regulates osteoblast proliferation, is mediated by VGCCs (verapamil inhibition); this elevated intracellular calcium ion concentrations and increased phospholipase A2 (PLA2) activity (bromophenyl bromide blockade). The increased PLA2 activity led to cyclooxygenase-dependent prostaglandin E2 synthesis (blocked by anti-inflammatory pain). Contrastingly, the elevated intracellular calcium ion concentrations led to CaM activation (blocked by N-(6-aminohexyl)-5-chloro-1-naphthalenesulphonamide hydrochloride, W-7). In addition, PEMFs promoted the differentiation of osteogenesis-associated cells by altering the Ca^2+^ oscillation pattern to resemble that of osteoblasts. Calcium oscillations can improve the efficiency and specificity of gene expression and direct cell differentiation.

High intracellular levels of Ca^2+^ can activate the NO/cyclic guanosine monophosphate (cGMP)/protein kinase G (PKG) pathway, owing to crosstalk between the Ca^2+^ and NO pathways (Jeandroz et al., 2013). Moreover, NO regulates intercellular information transfer and affects tissue blood flow. Thus, low NO levels promote osteoblast proliferation, whereas high concentrations inhibit the proliferation and differentiation of osteoblasts. Cheng et al. examined the effects of SEMF on osteogenesis through the NO-cGMP-PKG pathway by measuring ALP activity, Osterix (OSX) gene expression, and mineralised bone nodules. NOS activity was markedly higher than that in the control group after SEMF treatment. Additionally, OSX gene expression, ALP activity, and mineralised bone nodules were increased. Corresponding blockers were subsequently used to block the NO-cGMP-PKG pathway to determine whether SEMF-stimulated osteoblast maturation and mineralisation would be inhibited; the corresponding indices were all reduced (Cheng et al., 2011). As extracellular signals, electromagnetic signals belong to the first messengers of the cell and can act directly on VGCCs in the osteoblast membrane to increase Ca^2+^ inward flow. The inward-flowing Ca^2+^ combines with CaM to activate NOS, leading to increased NO production that consequently increases cGMP synthesis. Subsequently, cGMP activates PKG (Cheng et al., 2011; Pilla et al., 2011; Rangaswami et al., 2012; Zhong et al., 2012), which regulates gene transcription and mediates osteoblast proliferation and differentiation.

The MAPK signalling pathway transmits extracellular signals to the inside of the cell to control various cellular processes, such as proliferation, differentiation, migration, and death. This pathway is one of the important pathways through which electromagnetic fields cause the proliferation and differentiation of osteoblasts. Conventional MAPKs include ERK1/2, JNK, and p38. Yumoto et al. (2015) found that electromagnetic field radiation to osteoblast MC3T3-E1 cells induced the ERK1/2 and p38 MAPK pathways to enhance proliferation and upregulate the expression of various growth factors, including VEGF and platelet-derived growth factor. Ehnert et al. found that ELF-PEMFs increased total protein content and ALP activity and promoted the formation of a mineralised matrix by triggering the MAPK/ERK1/2 signalling pathway in osteoblasts. Similarly, inhibition of the ERK1/2 signalling pathway with U0126 prevented the activation of ALP activity and matrix mineralisation. In addition, the positive effects of ELF-PEMFs on osteoblast function were impaired (Ehnert et al., 2015). The increase in total protein content and ALP activity following ELF-PEMF treatment in osteoblasts was accompanied by a substantial upregulation of mitochondrial activity. This is consistent with a recent report that electromagnetic fields can promote osteogenic differentiation and bone anabolism by activating mitochondrial oxidative phosphorylation in bone progenitor cells and osteoblasts. However, the exact signalling pathway of their action is unknown (Hollenberg et al., 2021). In addition, PEMFs promote the expression of antioxidant enzymes (Poh et al., 2018) and enhance the activity of cytoprotective enzymes (Ehnert et al., 2017) through the MAPK/ERK signalling pathway, thereby shaping the microenvironment that promotes osteogenic differentiation.

TGF-β signalling pathways can promote osteoblast proliferation and early differentiation to osteoblast-like cells. These pathways are involved in PEMF-induced osteogenesis. BMP belongs to the TGF-β family and is a major factor inducing bone and cartilage formation *in vivo*. BMP initiates signalling cascade responses through typical Smad-dependent and atypical Smad-independent signalling pathways (Carreira et al., 2014; Salazar et al., 2016). Xie et al. found that PEMFs could activate the BMP-Smad1/5/8 signalling pathway by upregulating the expression of BMP II receptors on primary cilia, thereby promoting osteoblast differentiation and maturation in rats. Thus, the knockdown of BMP II receptors in osteoblasts reduces the promotion of osteogenic differentiation and maturation by PEMFs (Xie et al., 2016). *Smad7*, an antagonist of the TGF-β signalling pathway, is the putative target gene of miR21-5p, and PEMFs decreased *Smad7* expression. RUNX2 expression was increased by PEMF treatment. However, the miR21-5p inhibitor prevented the PEMF-induced RUNX2 expression in differentiating cells. These findings demonstrate that PEMFs regulate the expression of microRNA21 to activate TGF-β signalling and promote osteoblast differentiation (Selvamurugan et al., 2017).

The mTOR signalling pathway is an important molecular cascade involved in various physiological cellular processes, such as cell cycle and metabolic regulation, transcription, and translation, as well as cell differentiation and apoptosis. Activation of this pathway by PEMFs has been reported. Ferroni et al. (2018) revealed that PEMFs increase the expression of mTOR pathway-related proteins, such as AKT, MAPK kinase, and RRAGA. Furthermore, inhibitors of the mTOR pathway can reduce the osteogenic capacity of PEMFs. Considerable upregulation of the expression of bone-specific genes due to activation of Akt has been observed in cells after exposure to PEMFs at selected parameters (Poh et al., 2018).

The Notch signalling pathway is highly evolutionarily conserved. It is involved in important physiological activities, such as cell survival, proliferation, differentiation, and homeostatic regulation *in vivo*. When Notch receptors and their ligands interact, the Notch intracellular domain (NICD) is released and translocated to the nucleus to activate the transcription of target genes (Tian et al., 2017). The expression of the Notch receptor, its ligand DLL4, and target genes (*Hey1*, *Hes1*, and *Hes5*) was upregulated during PEMF-induced osteogenic differentiation of human bone marrow mesenchymal stem cells. Furthermore, application of Notch pathway inhibitors effectively suppressed the expression of osteogenic markers, including RUNX2, Dlx5, OSX, Hes1, and Hes5, which further explains the activation of the Notch pathway by PEMFs during osteogenesis (Bagheri et al., 2018).

#### 3.1.2 Osteoclasts

Physiological bone remodelling relies on a delicate equilibrium between osteoblast- and osteoclast-mediated bone formation and resorption, respectively. Osteoclasts, originating from the monocyte-macrophage lineage, exclusively regulate bone resorption. PEMFs can affect osteoclast function, thereby altering skeletal phenotypes. Moreover, they can impede the formation of osteoclasts, thereby reducing their numbers, downregulating the expression of osteoclast-related genes such as *TRAP* and *CTSK*, and diminishing the levels of inflammatory factors, including tumour necrosis factor-alpha and interleukin-1β. Additionally, PEMFs impact the differentiation and maturation of osteoclasts by inhibiting the activities of osteoclast transcription factors. Furthermore, they activate the T-cell nuclear factor and hinder the nuclear translocation of Ca^2+^ (Zhang et al., 2017; Song et al., 2018; Tschon et al., 2018; Noh et al., 2020). Insulin-like growth factor (IGF), the most abundant growth factor within the bone matrix, promotes osteoclast differentiation by regulating the expression of RANK and RANKL. Additionally, it facilitates the dynamic interaction between osteoblasts and osteoclasts, contributing to the maintenance of bone mass equilibrium during bone remodelling (Wang et al., 2006). Electromagnetic fields can markedly increase the mRNA expression level of IGF-1 in rat femur tissue *in vitro* to promote bone formation and inhibit bone resorption (Zhou et al., 2016). In addition, PEMFs at different frequencies can produce varying effects through different pathways: low-frequency stimulation enhances osteoclast differentiation and activity, mainly by activating the ERK and p38 MAPK pathways. Contrastingly, high-frequency stimulation inhibits osteoclast differentiation and reduces bone resorption through suppressed RANKL-induced phosphorylation of IκB (Hong et al., 2014). Hong et al. found that osteoclastogenic markers, such as NFATc1, TRAP, CTSK, MMP9, and DC-STAMP, were highly expressed at 7.5 Hz electromagnetic fields, whereas they were decreased at 45 Hz. Similarly, Nam found that electromagnetic fields at 10 G intensity and 40 Hz frequency reduced NFATc4 expression by decreasing TRPV1 and phosphorylated cyclic adenosine monophosphate-responsive element binding protein (CREB) levels, which regulated RANKL-induced osteoclast differentiation (Nam et al., 2023). Owing to the lack of clinical trial validation, the study of the mechanisms of different parameters is only at the theoretical stage. Thus, more valuable data could be obtained through clinical validation in the future.

### 3.2 Nerves

The nervous system regulates the development of various tissues, organs, and systems in the body. Bone tissue is also innervated by its corresponding peripheral nerves. Thus, the mechanisms underlying the promotion of fracture healing through the regulation of neuronal activity have garnered research interest. Most nerves in bone are neuropeptide-containing fibres, such as Aδ-fibres and C-fibres, which predominantly (>50%) express the calcitonin gene-related peptide (CGRP) and are sensitive to mechanical, chemical, and electrical stimuli (Lau et al., 2015; Yuen-Chi Lau et al., 2017; Brazill et al., 2019). CGRP, a 37-residue peptide produced by specific neurons through selective splicing of the calcitonin gene, is an important neuropeptide involved in bone growth and metabolism. It is produced in the sensory nerve fibres of bone tissue at the posterior root ganglion of the spinal cord and transported to the nerve endings to perform its *in vivo* regulatory and biological functions in the form of secretory granules (Tepper, 2018; Hendrikse et al., 2019). Clinical and experimental studies have confirmed that electromagnetic fields act on the peripheral nervous system to promote the biosynthesis and release of this peptide, which is involved in bone repair and regeneration (Naot et al., 2019; Mi et al., 2022). Electromagnetic field signals promote CGRP biosynthesis and release by activating the Ca^2+^/calmodulin-dependent protein kinase II/CREB signalling pathway. CGRP subsequently binds to target cell-specific G protein-coupled receptors to activate AC and elevate intracellular cAMP levels. Moreover, the cAMP-PKA signalling system mediates the essential pathway required to promote bone formation. Furthermore, the activation of PKA catalyses subunit phosphorylation and nuclear translocation, which phosphorylates CREB to activate the c-fos and c-jun families of transcription factors. This process ultimately enables the recognition of DNA-binding sites by Runx-2 and OCN in their promoter regions and accelerates osteoblast differentiation (Wang et al., 2019). This pathway also regulates the expression balance between important transcription factors for bone formation and resorption (e.g., RANKL, osteoprotegerin (OPG)), resulting in considerably reduced mRNA expression of RANKL and a substantial increase in the mRNA expression of OPG. This inhibits osteoclast formation and function and enhances fracture healing and bone metabolism (Villa et al., 2006; Ding et al., 2013; Zhang et al., 2016; Xu et al., 2020). The relationship between nerves and osteogenesis has been investigated from multiple perspectives. However, the acceleration of osteogenesis by electromagnetic fields through the neuronal secretion of CGRP warrants further exploration.

### 3.3 Blood vessels

Osteogenesis and angiogenesis, including cell-cell communication between vascular cells and osteoblasts, are essential for bone repair. VEGF is a highly specific vascular endothelial cell growth factor that binds to its receptor and activates a downstream signalling cascade, thereby controlling the survival, proliferation, and migration of vascular endothelial cells, which subsequently promotes neovascularisation and vascular permeability. VEGF contributes to endothelial mesenchymal stem cell aggregation into the vascular plexus, which plays a crucial role in neoangiogenesis and haemodialysis at the fracture site. H-type blood vessels can induce bone formation, and VEGF regulation of angiogenic processes has been closely linked to H-type blood vessels (Peng et al., 2020; Rodríguez-Merchán, 2021). In addition, electromagnetic field signals activate the VEGF signalling pathway to increase blood supply to the fracture region, thereby promoting bone repair (Hopper et al., 2009; Chen et al., 2018). Chen et al. (2018) showed that electromagnetic field signals activate VEGF receptors, leading to activated downstream components, such as PI3k/Akt, ERK1/2, and JNK. Furthermore, endothelial cell tubulogenesis was attenuated using inhibitors. Electromagnetic field-regulated angiogenesis during bone tissue regeneration can act through multiple pathways, including FGF, IGF, and platelet-derived growth factor pathways (Tepper et al., 2004; Chen et al., 2018; Yuan et al., 2018).

## 4 Clinical implications of PEMFs on bone healing

PEMFs affect biological tissues by generating electromagnetic fields of specific frequencies and intensities. They are widely used to treat various diseases and symptoms in clinical settings. Furthermore, their clinical application has extended to bone healing. In a randomised controlled trial, Shi et al. conducted a long-term follow-up on patients with fractures treated using PEMFs. Their findings indicated that early application of PEMF therapy significantly improves healing rates and reduces overall pain duration in patients with long bone fractures (Shi et al., 2013). Despite these positive outcomes, some studies have raised questions about the effectiveness of PEMF therapy. Hannemann et al. observed no significant differences in fracture healing in the PEMF group, sparking controversy over the therapeutic effects of this therapy (Hannemann et al., 2014). Furthermore, the assessment of clinical trial results on bone repair involving PEMFs raises concerns about the reliability of the outcomes. However, the inconsistency in PEMF parameters and treatment protocols used in different trials may affect the comparability of studies. Additionally, individual variations among patients and other factors before and after treatment may impact trial results. Thus, despite current research indicating the potential benefits of PEMFs in bone fracture repair, further standardised clinical trials are needed to confirm their efficacy. Furthermore, combining PEMF with other therapeutic interventions could synergistically enhance bone fracture repair outcomes. However, further in-depth research into the specific mechanisms and optimal application methods is required to fully leverage these synergistic effects. This could provide a foundation for optimised treatment strategies that better accommodate individual patient differences. Finally, existing research findings can facilitate the development of standardised guidelines for the use of PEMFs in bone repair, ensuring consistency and feasibility in clinical practice.

## 5 Limitations of PEMF therapy

The clinical application of PEMFs in bone repair has shown several limitations. Firstly, the diversity of treatment protocols poses a significant challenge. Variations in PEMF parameters, such as frequency, intensity, and treatment duration, in existing studies lead to inconsistency in optimal application methods. Moreover, the lack of standardised treatment protocols makes it difficult to compare different studies and formulate uniform treatment guidelines in clinical practice. Secondly, the variability in individual patient responses is a major issue. The physiological response of the human body to PEMFs may be influenced by factors such as age, sex, and comorbidities. However, there remains a lack of sufficient personalised research, making it challenging to accurately predict patient responses to PEMF therapy. This hinders the implementation of personalised treatment in clinical settings. Additionally, the demand for standardised guidelines highlights the knowledge gap in the use of PEMFs in bone repair treatment. The lack of clear guidelines for PEMF application makes it challenging for physicians to determine the optimal treatment approach in practical settings, thereby increasing uncertainty for both patients and medical institutions when choosing PEMF therapy. To overcome these obstacles, more large-scale studies, personalised research, and clear treatment guidelines are needed to advance the clinical application of PEMFs and maximise their potential in bone repair.

## 6 Conclusion

The active role of PEMFs in the treatment of bone-related diseases and their possible mechanisms have garnered considerable research interest. Some potential mechanisms underlying PEMF function have been elucidated, providing a theoretical and clinical basis for further application of electromagnetic fields to promote fracture healing. Electromagnetic fields can enhance the expression of bone-related genes and cellular activity by regulating ion channels and activating cellular signalling pathways. This ultimately alters the behaviour or function of osteoblasts to promote bone production and remodelling. However, the lack of consistent study parameters makes PEMF effects scientifically challenging to evaluate. Therefore, high-quality clinical studies and basic experiments are required to further clarify the optimal therapeutic parameters and molecular mechanisms of PEMFs. Such in-depth investigation could ensure the optimisation of electromagnetic fields to a more effective and accurate alternative therapy for the treatment of bone disease and regeneration.

## References

[B1] Atalay Y. Gunes N. Guner M. D. Akpolat V. Celik M. S. Guner R. (2015). Pentoxifylline and electromagnetic field improved bone fracture healing in rats. Drug Des. Devel Ther. 9, 5195–5201. 10.2147/dddt.S89669 PMC4571933 26388687

[B2] Aydin N. Bezer M. (2011). The effect of an intramedullary implant with a static magnetic field on the healing of the osteotomised rabbit femur. Int. Orthop. 35 (1), 135–141. 10.1007/s00264-009-0932-9 20062989 PMC3014488

[B3] Bagheri L. Pellati A. Rizzo P. Aquila G. Massari L. De Mattei M. (2018). Notch pathway is active during osteogenic differentiation of human bone marrow mesenchymal stem cells induced by pulsed electromagnetic fields. J. Tissue Eng. Regen. Med. 12 (2), 304–315. 10.1002/term.2455 28482141

[B4] Barnaba S. Papalia R. Ruzzini L. Sgambato A. Maffulli N. Denaro V. (2013). Effect of pulsed electromagnetic fields on human osteoblast cultures. Physiother. Res. Int. 18 (2), 109–114. 10.1002/pri.1536 22991203

[B5] Barnaba S. A. Ruzzini L. Di Martino A. Lanotte A. Sgambato A. Denaro V. (2012). Clinical significance of different effects of static and pulsed electromagnetic fields on human osteoclast cultures. Rheumatol. Int. 32 (4), 1025–1031. 10.1007/s00296-010-1724-7 21246371

[B6] Benazzo F. Cadossi M. Cavani F. Fini M. Giavaresi G. Setti S. (2008). Cartilage repair with osteochondral autografts in sheep: effect of biophysical stimulation with pulsed electromagnetic fields. J. Orthop. Res. 26 (5), 631–642. 10.1002/jor.20530 18176941

[B7] Bique A. M. Kaivosoja E. Mikkonen M. Paulasto-Kröckel M. (2016). Choice of osteoblast model critical for studying the effects of electromagnetic stimulation on osteogenesis *in vitro* . Electromagn. Biol. Med. 35 (4), 353–364. 10.3109/15368378.2016.1138124 27355896

[B8] Bodamyali T. Bhatt B. Hughes F. J. Winrow V. R. Kanczler J. M. Simon B. (1998). Pulsed electromagnetic fields simultaneously induce osteogenesis and upregulate transcription of bone morphogenetic proteins 2 and 4 in rat osteoblasts *in vitro* . Biochem. Biophys. Res. Commun. 250 (2), 458–461. 10.1006/bbrc.1998.9243 9753652

[B9] Brazill J. M. Beeve A. T. Craft C. S. Ivanusic J. J. Scheller E. L. (2019). Nerves in bone: evolving concepts in pain and anabolism. J. Bone Min. Res. 34 (8), 1393–1406. 10.1002/jbmr.3822 PMC6697229 31247122

[B10] Buzzá E. P. Shibli J. A. Barbeiro R. H. Barbosa J. R. (2003). Effects of electromagnetic field on bone healing around commercially pure titanium surface: histologic and mechanical study in rabbits. Implant Dent. 12 (2), 182–187. 10.1097/01.id.0000058385.23346.4d 12861888

[B11] Cadossi R. Massari L. Racine-Avila J. Aaron R. K. (2020). Pulsed electromagnetic field stimulation of bone healing and joint preservation: cellular mechanisms of skeletal response. J. Am. Acad. Orthop. Surg. Glob. Res. Rev. 4 (5), e1900155. 10.5435/JAAOSGlobal-D-19-00155 33970582 PMC7434032

[B12] Cai K. Jiao Y. Quan Q. Hao Y. Liu J. Wu L. (2021). Improved activity of MC3T3-E1 cells by the exciting piezoelectric BaTiO(3)/TC4 using low-intensity pulsed ultrasound. Bioact. Mater 6 (11), 4073–4082. 10.1016/j.bioactmat.2021.04.016 33997494 PMC8090998

[B13] Canè V. Botti P. Farneti D. Soana S. (1991). Electromagnetic stimulation of bone repair: a histomorphometric study. J. Orthop. Res. 9 (6), 908–917. 10.1002/jor.1100090618 1919855

[B14] Carreira A. C. Lojudice F. H. Halcsik E. Navarro R. D. Sogayar M. C. Granjeiro J. M. (2014). Bone morphogenetic proteins: facts, challenges, and future perspectives. J. Dent. Res. 93 (4), 335–345. 10.1177/0022034513518561 24389809

[B15] Chalidis B. Sachinis N. Assiotis A. Maccauro G. Graziani F. (2011). Stimulation of bone formation and fracture healing with pulsed electromagnetic fields: biologic responses and clinical implications. Int. J. Immunopathol. Pharmacol. 24 (1 Suppl. 2), 17–20. 10.1177/03946320110241s204 21669132

[B16] Chang K. Chang W. H. Huang S. Huang S. Shih C. (2005). Pulsed electromagnetic fields stimulation affects osteoclast formation by modulation of osteoprotegerin, RANK ligand and macrophage colony-stimulating facto. J. Orthop. Res. 23 (6), 1308–1314. 10.1016/j.orthres.2005.03.012.1100230611 15913941

[B17] Chang K. Chang W. H. Tsai M. T. Shih C. (2006). Pulsed electromagnetic fields accelerate apoptotic rate in osteoclasts. Connect. Tissue Res. 47 (4), 222–228. 10.1080/03008200600858783 16987754

[B18] Chang K. Hong-Shong Chang W. Yu Y. H. Shih C. (2004a). Pulsed electromagnetic field stimulation of bone marrow cells derived from ovariectomized rats affects osteoclast formation and local factor production. Bioelectromagnetics 25 (2), 134–141. 10.1002/bem.10168 14735564

[B19] Chang W. H. Chen L. T. Sun J. S. Lin F. H. (2004b). Effect of pulse-burst electromagnetic field stimulation on osteoblast cell activities. Bioelectromagnetics 25 (6), 457–465. 10.1002/bem.20016 15300732

[B20] Chen Y. Ye L. Guan L. Fan P. Liu R. Liu H. (2018). Physiological electric field works via the VEGF receptor to stimulate neovessel formation of vascular endothelial cells in a 3D environment. Biol. Open 7 (9), bio035204. 10.1242/bio.035204 30232195 PMC6176943

[B21] Cheng G. Zhai Y. Chen K. Zhou J. Han G. Zhu R. (2011). Sinusoidal electromagnetic field stimulates rat osteoblast differentiation and maturation via activation of NO-cGMP-PKG pathway. Nitric Oxide 25 (3), 316–325. 10.1016/j.niox.2011.05.009 21664476

[B22] Chiu K. H. Ou K. L. Lee S. Y. Lin C. T. Chang W. J. Chen C. C. (2007). Static magnetic fields promote osteoblast-like cells differentiation via increasing the membrane rigidity. Ann. Biomed. Eng. 35 (11), 1932–1939. 10.1007/s10439-007-9370-2 17721730

[B23] Clark C. C. Wang W. Brighton C. T. (2014). Up-regulation of expression of selected genes in human bone cells with specific capacitively coupled electric fields. J. Orthop. Res. 32 (7), 894–903. 10.1002/jor.22595 24644137

[B24] Collon K. Gallo M. C. Lieberman J. R. (2021). Musculoskeletal tissue engineering: regional gene therapy for bone repair. Biomaterials 275, 120901. 10.1016/j.biomaterials.2021.120901 34091300

[B25] Cook J. J. Summers N. J. Cook E. A. (2015). Healing in the new millennium: bone stimulators. Clin. Podiatr. Med. Surg. 32 (1), 45–59. 10.1016/j.cpm.2014.09.003 25440417

[B26] Dalisson B. Charbonnier B. Aoude A. Gilardino M. Harvey E. Makhoul N. (2021). Skeletal regeneration for segmental bone loss: vascularised grafts, analogues and surrogates. Acta Biomater. 136, 37–55. 10.1016/j.actbio.2021.09.053 34626818

[B27] Denaro V. Cittadini A. Barnaba S. A. Ruzzini L. Denaro L. Rettino A. (2008). Static electromagnetic fields generated by corrosion currents inhibit human osteoblast differentiation. Spine (Phila Pa 1976) 33 (9), 955–959. 10.1097/BRS.0b013e31816c90b8 18427315

[B28] deVet T. Jhirad A. Pravato L. Wohl G. R. (2021). Bone bioelectricity and bone-cell response to electrical stimulation: a review. Crit. Rev. Biomed. Eng. 49 (1), 1–19. 10.1615/CritRevBiomedEng.2021035327 34347984

[B29] Ding L. Song T. Yi C. Huang Y. Yu W. Ling L. (2013). Transcutaneous electrical nerve stimulation (TENS) improves the diabetic cytopathy (DCP) via up-regulation of CGRP and cAMP. PLoS One 8 (2), e57477. 10.1371/journal.pone.0057477 23468996 PMC3585412

[B30] Ehnert S. Falldorf K. Fentz A. K. Ziegler P. Schröter S. Freude T. (2015). Primary human osteoblasts with reduced alkaline phosphatase and matrix mineralization baseline capacity are responsive to extremely low frequency pulsed electromagnetic field exposure - clinical implication possible. Bone Rep. 3, 48–56. 10.1016/j.bonr.2015.08.002 28377966 PMC5365212

[B31] Ehnert S. Fentz A. K. Schreiner A. Birk J. Wilbrand B. Ziegler P. (2017). Extremely low frequency pulsed electromagnetic fields cause antioxidative defense mechanisms in human osteoblasts via induction of •O(2)(-) and H(2)O(2). Sci. Rep. 7 (1), 14544. 10.1038/s41598-017-14983-9 29109418 PMC5673962

[B32] Eischen-Loges M. Oliveira K. M. C. Bhavsar M. B. Barker J. H. Leppik L. (2018). Pretreating mesenchymal stem cells with electrical stimulation causes sustained long-lasting pro-osteogenic effects. PeerJ 6, e4959. 10.7717/peerj.4959 29910982 PMC6001709

[B33] El-Rashidy A. A. Roether J. A. Harhaus L. Kneser U. Boccaccini A. R. (2017). Regenerating bone with bioactive glass scaffolds: a review of *in vivo* studies in bone defect models. Acta Biomater. 62, 1–28. 10.1016/j.actbio.2017.08.030 28844964

[B34] Ercan B. Webster T. J. (2008). Greater osteoblast proliferation on anodized nanotubular titanium upon electrical stimulation. Int. J. Nanomedicine 3 (4), 477–485. 10.2147/ijn.s3780 19337416 PMC2636582

[B35] Esmail M. Y. Sun L. Yu L. Xu H. Shi L. Zhang J. (2012). Effects of PEMF and glucocorticoids on proliferation and differentiation of osteoblasts. Electromagn. Biol. Med. 31 (4), 375–381. 10.3109/15368378.2012.662196 22676065

[B36] Fathi E. Farahzadi R. (2017). Enhancement of osteogenic differentiation of rat adipose tissue-derived mesenchymal stem cells by zinc sulphate under electromagnetic field via the PKA, ERK1/2 and Wnt/β-catenin signaling pathways. PLoS One 12 (3), e0173877. 10.1371/journal.pone.0173877 28339498 PMC5365128

[B37] Ferroni L. Gardin C. Dolkart O. Salai M. Barak S. Piattelli A. (2018). Pulsed electromagnetic fields increase osteogenetic commitment of MSCs via the mTOR pathway in TNF-α mediated inflammatory conditions: an *in-vitro* study. Sci. Rep. 8 (1), 5108. 10.1038/s41598-018-23499-9 29572540 PMC5865106

[B38] Fredericks D. C. Nepola J. V. Baker J. T. Abbott J. Simon B. (2000). Effects of pulsed electromagnetic fields on bone healing in a rabbit tibial osteotomy model. J. Orthop. Trauma 14 (2), 93–100. 10.1097/00005131-200002000-00004 10716379

[B39] Fredericks D. C. Petersen E. B. Rhodes M. Larew G. A. Nepola J. V. (2019). The effect of pulsed electromagnetic field and combined magnetic field exposure time on healing of a rabbit tibial osteotomy. Iowa Orthop. J. 39 (2), 20–26. 32577103 PMC7047300

[B40] Fredericks D. C. Piehl D. J. Baker J. T. Abbott J. Nepola J. V. (2003). Effects of pulsed electromagnetic field stimulation on distraction osteogenesis in the rabbit tibial leg lengthening model. J. Pediatr. Orthop. 23 (4), 478–483. 10.1097/01241398-200307000-00012 12826946

[B41] Grana D. R. Marcos H. J. Kokubu G. A. (2008). Pulsed electromagnetic fields as adjuvant therapy in bone healing and peri-implant bone formation: an experimental study in rats. Acta Odontol. Latinoam. 21 (1), 77–83. 18841750

[B42] Guizzardi S. Di Silvestre M. Govoni P. Scandroglio R. (1994). Pulsed electromagnetic field stimulation on posterior spinal fusions: a histological study in rats. J. Spinal Disord. 7 (1), 36–40. 10.1097/00002517-199407010-00005 8186587

[B43] Hannemann P. F. van Wezenbeek M. R. Kolkman K. A. Twiss E. L. Berghmans C. H. Dirven P. A. (2014). CT scan-evaluated outcome of pulsed electromagnetic fields in the treatment of acute scaphoid fractures: a randomised, multicentre, double-blind, placebo-controlled trial. Bone Jt. J. 96-b (8), 1070–1076. 10.1302/0301-620x.96b8.33767 25086123

[B44] He J. Zhang Y. Chen J. Zheng S. Huang H. Dong X. (2015). Effects of pulsed electromagnetic fields on the expression of NFATc1 and CAII in mouse osteoclast-like cells. Aging Clin. Exp. Res. 27 (1), 13–19. 10.1007/s40520-014-0239-6 24869857

[B45] He M. Wang Q. Feng Y. Gao X. He C. Li J. (2022). Spatiotemporal management of the osteoimmunomodulation of fibrous scaffolds by loading a novel amphiphilic nanomedicine. ACS Appl. Mater Interfaces 14 (12), 13991–14003. 10.1021/acsami.1c20809 35311248

[B46] He Z. Selvamurugan N. Warshaw J. Partridge N. C. (2018). Pulsed electromagnetic fields inhibit human osteoclast formation and gene expression via osteoblasts. Bone 106, 194–203. 10.1016/j.bone.2017.09.020 28965919

[B47] Heermeier K. Spanner M. Träger J. Gradinger R. Strauss P. G. Kraus W. (1998). Effects of extremely low frequency electromagnetic field (EMF) on collagen type I mRNA expression and extracellular matrix synthesis of human osteoblastic cells. Bioelectromagnetics 19 (4), 222–231. 10.1002/(sici)1521-186x(1998)19:4<222::aid-bem4>3.0.co;2-3 9581965

[B48] Hendrikse E. R. Bower R. L. Hay D. L. Walker C. S. (2019). Molecular studies of CGRP and the CGRP family of peptides in the central nervous system. Cephalalgia 39 (3), 403–419. 10.1177/0333102418765787 29566540

[B49] Hiemer B. Ziebart J. Jonitz-Heincke A. Grunert P. C. Su Y. Hansmann D. (2016). Magnetically induced electrostimulation of human osteoblasts results in enhanced cell viability and osteogenic differentiation. Int. J. Mol. Med. 38 (1), 57–64. 10.3892/ijmm.2016.2590 27220915 PMC4899037

[B50] Hofmann A. Gorbulev S. Guehring T. Schulz A. P. Schupfner R. Raschke M. (2020). Autologous iliac bone graft compared with biphasic hydroxyapatite and calcium sulfate cement for the treatment of bone defects in tibial plateau fractures: a prospective, randomized, open-label, multicenter study. J. Bone Jt. Surg. Am. 102 (3), 179–193. 10.2106/jbjs.19.00680 PMC7508276 31809394

[B51] Hollenberg A. M. Huber A. Smith C. O. Eliseev R. A. (2021). Electromagnetic stimulation increases mitochondrial function in osteogenic cells and promotes bone fracture repair. Sci. Rep. 11 (1), 19114. 10.1038/s41598-021-98625-1 34580378 PMC8476611

[B52] Hong J. M. Kang K. S. Yi H. G. Kim S. Y. Cho D. W. (2014). Electromagnetically controllable osteoclast activity. Bone 62, 99–107. 10.1016/j.bone.2014.02.005 24556539

[B53] Hopper R. A. VerHalen J. P. Tepper O. Mehrara B. J. Detch R. Chang E. I. (2009). Osteoblasts stimulated with pulsed electromagnetic fields increase HUVEC proliferation via a VEGF-A independent mechanism. Bioelectromagnetics 30 (3), 189–197. 10.1002/bem.20459 19194859

[B54] Hou J. Luo T. Chen S. Lin S. Yang M. M. Li G. (2019). Calcium spike patterns reveal linkage of electrical stimulus and MSC osteogenic differentiation. IEEE Trans. Nanobioscience 18 (1), 3–9. 10.1109/tnb.2018.2881004 30442614

[B55] Huang C. P. Chen X. M. Chen Z. Q. (2008). Osteocyte: the impresario in the electrical stimulation for bone fracture healing. Med. Hypotheses 70 (2), 287–290. 10.1016/j.mehy.2007.05.044 17689020

[B56] Inoue N. Ohnishi I. Chen D. Deitz L. W. Schwardt J. D. Chao E. Y. (2002). Effect of pulsed electromagnetic fields (PEMF) on late-phase osteotomy gap healing in a canine tibial model. J. Orthop. Res. 20 (5), 1106–1114. 10.1016/s0736-0266(02)00031-1 12382979

[B57] Isaacson B. M. Bloebaum R. D. (2010). Bone bioelectricity: what have we learned in the past 160 years? J. Biomed. Mater Res. A 95 (4), 1270–1279. 10.1002/jbm.a.32905 20878899

[B58] Jeandroz S. Lamotte O. Astier J. Rasul S. Trapet P. Besson-Bard A. (2013). There's more to the picture than meets the eye: nitric oxide cross talk with Ca2+ signaling. Plant Physiol. 163 (2), 459–470. 10.1104/pp.113.220624 23749853 PMC3793028

[B59] Jing D. Zhai M. Tong S. Xu F. Cai J. Shen G. (2016). Pulsed electromagnetic fields promote osteogenesis and osseointegration of porous titanium implants in bone defect repair through a Wnt/β-catenin signaling-associated mechanism. Sci. Rep. 6, 32045. 10.1038/srep32045 27555216 PMC4995433

[B60] Kahanovitz N. Arnoczky S. P. Nemzek J. A. Shores A. J. S. (1994). The effect of electromagnetic pulsing on posterior lumbar spinal fusions in dogs. Spine (Phila Pa 1976) 19 6, 705–709. 10.1097/00007632-199403001-00010 8009336

[B61] Khalifeh J. M. Zohny Z. MacEwan M. Stephen M. Johnston W. Gamble P. (2018). Electrical stimulation and bone healing: a review of current technology and clinical applications. IEEE Rev. Biomed. Eng. 11, 217–232. 10.1109/rbme.2018.2799189 29994564

[B62] Kim D. Lee B. Marshall B. P. Jang E. Thomopoulos S. Jun Y. S. (2020). Pulsed electrical stimulation enhances body fluid transport for collagen biomineralization. ACS Appl. Bio Mater 3 (2), 902–910. 10.1021/acsabm.9b00979 35019292

[B63] Kim E. C. Park J. Kwon I. K. Lee S. W. Park S. J. Ahn S. J. (2017). Static magnetic fields promote osteoblastic/cementoblastic differentiation in osteoblasts, cementoblasts, and periodontal ligament cells. J. Periodontal Implant Sci. 47 (5), 273–291. 10.5051/jpis.2017.47.5.273 29093986 PMC5663666

[B64] Kim I. S. Song J. K. Song Y. M. Cho T. H. Lee T. H. Lim S. S. (2009). Novel effect of biphasic electric current on *in vitro* osteogenesis and cytokine production in human mesenchymal stromal cells. Tissue Eng. Part A 15 (9), 2411–2422. 10.1089/ten.tea.2008.0554 19292669

[B65] Kim I. S. Song J. K. Zhang Y. L. Lee T. H. Cho T. H. Song Y. M. (2006). Biphasic electric current stimulates proliferation and induces VEGF production in osteoblasts. Biochim. Biophys. Acta 1763 (9), 907–916. 10.1016/j.bbamcr.2006.06.007 16930744

[B66] Kold S. E. Hickman J. Meisen F. (1987). Preliminary study of quantitative aspects and the effect of pulsed electromagnetic field treatment on the incorporation of equine cancellous bone grafts. Equine Vet. J. 19 (2), 120–124. 10.1111/j.2042-3306.1987.tb02603.x 3552658

[B67] Kooistra B. W. Jain A. Hanson B. P. (2009). Electrical stimulation: nonunions. Indian J. Orthop. 43 (2), 149–155. 10.4103/0019-5413.50849 19838363 PMC2762246

[B68] Lau Y. C. Qian X. Po K. T. Li L. M. Guo X. (2015). Electrical stimulation at the dorsal root ganglion preserves trabecular bone mass and microarchitecture of the tibia in hindlimb-unloaded rats. Osteoporos. Int. 26 (2), 481–488. 10.1007/s00198-014-2866-3 25212672

[B69] Law H. T. Annan I. McCarthy I. D. Hughes S. P. Stead A. C. Camburn M. A. (1985). The effect of induced electric currents on bone after experimental osteotomy in sheep. J. Bone Jt. Surg. Br. 67 (3), 463–469. 10.1302/0301-620x.67b3.3873459 3873459

[B70] Leppik L. Bhavsar M. B. Oliveira K. M. C. Eischen-Loges M. Mobini S. Barker J. H. (2019). Construction and use of an electrical stimulation chamber for enhancing osteogenic differentiation in mesenchymal stem/stromal cells *in vitro* . J. Vis. Exp. 143. 10.3791/59127 30774122

[B71] Leppik L. Oliveira K. M. C. Bhavsar M. B. Barker J. H. (2020). Electrical stimulation in bone tissue engineering treatments. Eur. J. Trauma Emerg. Surg. 46 (2), 231–244. 10.1007/s00068-020-01324-1 32078704 PMC7113220

[B72] Li Y. Hoffman M. D. Benoit D. S. W. (2021). Matrix metalloproteinase (MMP)-degradable tissue engineered periosteum coordinates allograft healing via early stage recruitment and support of host neurovasculature. Biomaterials 268, 120535. 10.1016/j.biomaterials.2020.120535 33271450 PMC8110201

[B73] Lin H. Y. Lin Y. J. (2011). *In vitro* effects of low frequency electromagnetic fields on osteoblast proliferation and maturation in an inflammatory environment. Bioelectromagnetics 32 (7), 552–560. 10.1002/bem.20668 21448989

[B74] Lirani-Galvão A. P. Bergamaschi C. T. Silva O. L. Lazaretti-Castro M. (2006). Electrical field stimulation improves bone mineral density in ovariectomized rats. Braz J. Med. Biol. Res. 39 (11), 1501–1505. 10.1590/s0100-879x2006001100014 17146563

[B75] Martin-Granados C. McCaig C. D. (2014). Harnessing the electric spark of life to cure skin wounds. Adv. Wound Care (New Rochelle) 3 (2), 127–138. 10.1089/wound.2013.0451 24761353 PMC3928811

[B76] Matsumoto H. Ochi M. Abiko Y. Hirose Y. Kaku T. Sakaguchi K. (2000). Pulsed electromagnetic fields promote bone formation around dental implants inserted into the femur of rabbits. Clin. Oral Implants Res. 11 (4), 354–360. 10.1034/j.1600-0501.2000.011004354.x 11168228

[B77] Maziarz A. Kocan B. Bester M. Budzik S. Cholewa M. Ochiya T. (2016). How electromagnetic fields can influence adult stem cells: positive and negative impacts. Stem Cell Res. Ther. 7 (1), 54. 10.1186/s13287-016-0312-5 27086866 PMC4834823

[B78] Mi J. Xu J. K. Yao Z. Yao H. Li Y. He X. (2022). Implantable electrical stimulation at dorsal root ganglions accelerates osteoporotic fracture healing via calcitonin gene-related peptide. Adv. Sci. (Weinh) 9 (1), e2103005. 10.1002/advs.202103005 34708571 PMC8728818

[B79] Muhsin A. U. Islam K. M. Ahmed A. M. Islam M. S. Rabbani K. S. Rahman S. M. (1991). Effect of pulsed electromagnetic field on healing of experimental nonunion in rat tibiae. Bangladesh Med. Res. Counc. Bull. 17 (1), 1–10. 1953591

[B80] Nam M. H. Park H. J. Seo Y. K. (2023). Reduction of osteoclastic differentiation of raw 264.7 cells by EMF exposure through TRPV4 and p-CREB pathway. Int. J. Mol. Sci. 24 (4), 3058. 10.3390/ijms24043058 36834470 PMC9959640

[B81] Naot D. Musson D. S. Cornish J. (2019). The activity of peptides of the calcitonin family in bone. Physiol. Rev. 99 (1), 781–805. 10.1152/physrev.00066.2017 30540227

[B82] Noh J. Y. Yang Y. Jung H. (2020). Molecular mechanisms and emerging therapeutics for osteoporosis. Int. J. Mol. Sci. 21 (20), 7623. 10.3390/ijms21207623 33076329 PMC7589419

[B83] Norton L. A. Rodan G. A. Bourret L. A. (1977). Epiphyseal cartilage cAMP changes produced by electrical and mechanical perturbations. Clin. Orthop. Relat. Res. 124, 59–68. 10.1097/00003086-197705000-00009 202424

[B84] Oltean-Dan D. Dogaru G. B. Apostu D. Mester A. Benea H. R. C. Paiusan M. G. (2019). Enhancement of bone consolidation using high-frequency pulsed electromagnetic fields (HF-PEMFs): an experimental study on rats. Bosn. J. Basic Med. Sci. 19 (2), 201–209. 10.17305/bjbms.2019.3854 30794499 PMC6535386

[B85] Ortman L. F. Casey D. M. Deers M. (1992). Bioelectric stimulation and residual ridge resorption. J. Prosthet. Dent. 67 (1), 67–71. 10.1016/0022-3913(92)90052-c 1548612

[B86] Ottani V. Raspanti M. Martini D. Tretola G. Ruggeri A. Jr. Franchi M. (2002). Electromagnetic stimulation on the bone growth using backscattered electron imaging. Micron 33 (2), 121–125. 10.1016/s0968-4328(01)00008-7 11567880

[B87] Pall M. L. (2013). Electromagnetic fields act via activation of voltage-gated calcium channels to produce beneficial or adverse effects. J. Cell Mol. Med. 17 (8), 958–965. 10.1111/jcmm.12088 23802593 PMC3780531

[B88] Peng Y. Wu S. Li Y. Crane J. L. (2020). Type H blood vessels in bone modeling and remodeling. Theranostics 10 (1), 426–436. 10.7150/thno.34126 31903130 PMC6929606

[B89] Pettersen E. Shah F. A. Ortiz-Catalan M. (2021). Enhancing osteoblast survival through pulsed electrical stimulation and implications for osseointegration. Sci. Rep. 11 (1), 22416. 10.1038/s41598-021-01901-3 34789829 PMC8599699

[B90] Pilla A. Fitzsimmons R. Muehsam D. Wu J. Rohde C. Casper D. (2011). Electromagnetic fields as first messenger in biological signaling: application to calmodulin-dependent signaling in tissue repair. Biochim. Biophys. Acta 1810 (12), 1236–1245. 10.1016/j.bbagen.2011.10.001 22005645

[B91] Poh P. S. P. Seeliger C. Unger M. Falldorf K. Balmayor E. R. van Griensven M. (2018). Osteogenic effect and cell signaling activation of extremely low-frequency pulsed electromagnetic fields in adipose-derived mesenchymal stromal cells. Stem Cells Int. 2018, 1–11. 10.1155/2018/5402853 PMC6079332 30123287

[B92] Puricelli E. Dutra N. B. Ponzoni D. (2009). Histological evaluation of the influence of magnetic field application in autogenous bone grafts in rats. Head. Face Med. 5, 1. 10.1186/1746-160x-5-1 19134221 PMC2635355

[B93] Qi Z. Xia P. Pan S. Zheng S. Fu C. Chang Y. (2018). Combined treatment with electrical stimulation and insulin-like growth factor-1 promotes bone regeneration *in vitro* . PLoS One 13 (5), e0197006. 10.1371/journal.pone.0197006 29746517 PMC5944947

[B94] Qiu X. S. Li X. G. Chen Y. X. (2020). Pulsed electromagnetic field (PEMF): a potential adjuvant treatment for infected nonunion. Med. Hypotheses 136, 109506. 10.1016/j.mehy.2019.109506 31841766

[B95] Rangaswami H. Schwappacher R. Tran T. Chan G. C. Zhuang S. Boss G. R. (2012). Protein kinase G and focal adhesion kinase converge on src/akt/β-catenin signaling module in osteoblast mechanotransduction. J. Biol. Chem. 287 (25), 21509–21519. 10.1074/jbc.M112.347245 22563076 PMC3375572

[B96] Reid B. Zhao M. (2014). The electrical response to injury: molecular mechanisms and wound healing. Adv. Wound Care (New Rochelle) 3 (2), 184–201. 10.1089/wound.2013.0442 24761358 PMC3928722

[B97] Rodríguez-Merchán E. C. (2021). A review of recent developments in the molecular mechanisms of bone healing. Int. J. Mol. Sci. 22 (2), 767. 10.3390/ijms22020767 33466612 PMC7828700

[B98] Salazar V. S. Gamer L. W. Rosen V. (2016). BMP signalling in skeletal development, disease and repair. Nat. Rev. Endocrinol. 12 (4), 203–221. 10.1038/nrendo.2016.12 26893264

[B99] Sanders-Shamis M. Bramlage L. R. Weisbrode S. E. Gabel A. A. (1989). A preliminary investigation of the effect of selected electromagnetic field devices on healing of cannon bone osteotomies in horses. Equine Vet. J. 21 (3), 201–205. 10.1111/j.2042-3306.1989.tb02145.x 2731509

[B100] Sarker A. B. Nashimuddin A. N. Islam K. M. Rabbani K. S. Rahman M. Mushin A. U. (1993). Effect of PEMF on fresh fracture-healing in rat tibia. Bangladesh Med. Res. Counc. Bull. 19 (3), 103–112. 8031284

[B101] Selvamurugan N. He Z. Rifkin D. Dabovic B. Partridge N. C. (2017). Pulsed electromagnetic field regulates MicroRNA 21 expression to activate TGF-*β* signaling in human bone marrow stromal cells to enhance osteoblast differentiation. Stem Cells Int. 2017, 1–17. 10.1155/2017/2450327 PMC5420424 28512472

[B102] Shao J. Li Z. Zhou J. Li K. Qin R. Chen K. (2019). Effect of low-frequency pulsed electromagnetic fields on activity of rat calvarial osteoblasts through IGF-1R/NO signaling pathway. Zhejiang Da Xue Xue Bao Yi Xue Ban. 48 (2), 158–164. 10.3785/j.issn.1008-9292.2019.04.06 31309753 PMC8800640

[B103] Shen W. W. Zhao J. H. (2010). Pulsed electromagnetic fields stimulation affects BMD and local factor production of rats with disuse osteoporosis. Bioelectromagnetics 31 (2), 113–119. 10.1002/bem.20535 19670410

[B104] Shi H. F. Xiong J. Chen Y. X. Wang J. F. Qiu X. S. Wang Y. H. (2013). Early application of pulsed electromagnetic field in the treatment of postoperative delayed union of long-bone fractures: a prospective randomized controlled study. BMC Musculoskelet. Disord. 14, 35. 10.1186/1471-2474-14-35 23331333 PMC3556314

[B105] Shimizu E. Matsuda-Honjyo Y. Samoto H. Saito R. Nakajima Y. Nakayama Y. (2004). Static magnetic fields-induced bone sialoprotein (BSP) expression is mediated through FGF2 response element and pituitary-specific transcription factor-1 motif. J. Cell Biochem. 91 (6), 1183–1196. 10.1002/jcb.20002 15048873

[B106] Shuai C. Yang W. Peng S. Gao C. Guo W. Lai Y. (2018). Physical stimulations and their osteogenesis-inducing mechanisms. Int. J. Bioprint 4 (2), 138. 10.18063/IJB.v4i2.138 33102916 PMC7581999

[B107] Smith R. L. Nagel D. A. (1983). Effects of pulsing electromagnetic fields on bone growth and articular cartilage. Clin. Orthop. Relat. Res. 181, 277–282. 10.1097/00003086-198312000-00043 6641061

[B108] Soda A. Ikehara T. Kinouchi Y. Yoshizaki K. (2008). Effect of exposure to an extremely low frequency-electromagnetic field on the cellular collagen with respect to signaling pathways in osteoblast-like cells. J. Med. Invest. 55 (3-4), 267–278. 10.2152/jmi.55.267 18797142

[B109] Song Z. H. Xie W. Zhu S. Y. Pan J. J. Zhou L. Y. He C. Q. (2018). Effects of PEMFs on Osx, Ocn, TRAP, and CTSK gene expression in postmenopausal osteoporosis model mice. Int. J. Clin. Exp. Pathol. 11 (3), 1784–1790. 31938285 PMC6958114

[B110] Sun L. Y. Hsieh D. K. Lin P. C. Chiu H. T. Chiou T. W. (2010). Pulsed electromagnetic fields accelerate proliferation and osteogenic gene expression in human bone marrow mesenchymal stem cells during osteogenic differentiation. Bioelectromagnetics 31 (3), 209–219. 10.1002/bem.20550 19866474

[B111] Sundelacruz S. Levin M. Kaplan D. L. (2008). Membrane potential controls adipogenic and osteogenic differentiation of mesenchymal stem cells. PLoS One 3 (11), e3737. 10.1371/journal.pone.0003737 19011685 PMC2581599

[B112] Takano-Yamamoto T. Kawakami M. Sakuda M. (1992). Effect of a pulsing electromagnetic field on demineralized bone-matrix-induced bone formation in a bony defect in the premaxilla of rats. J. Dent. Res. 71 (12), 1920–1925. 10.1177/00220345920710121301 1452895

[B113] Taylor B. C. French B. G. Fowler T. T. Russell J. Poka A. (2012). Induced membrane technique for reconstruction to manage bone loss. J. Am. Acad. Orthop. Surg. 20 (3), 142–150. 10.5435/jaaos-20-03-142 22382286

[B114] Taylor K. F. Inoue N. Rafiee B. Tis J. E. McHale K. A. Chao E. Y. (2006). Effect of pulsed electromagnetic fields on maturation of regenerate bone in a rabbit limb lengthening model. J. Orthop. Res. 24 (1), 2–10. 10.1002/jor.20014 16419963

[B115] Tepper O. M. Callaghan M. J. Chang E. I. Galiano R. D. Bhatt K. A. Baharestani S. (2004). Electromagnetic fields increase *in vitro* and *in vivo* angiogenesis through endothelial release of FGF-2. Faseb J. 18 (11), 1231–1233. 10.1096/fj.03-0847fje 15208265

[B116] Tepper S. J. (2018). History and review of anti-calcitonin gene-related peptide (CGRP) therapies: from translational research to treatment. Headache 58 (Suppl. 3), 238–275. 10.1111/head.13379 30242830

[B117] Tian Y. Xu Y. Xue T. Chen L. Shi B. Shu B. (2017). Notch activation enhances mesenchymal stem cell sheet osteogenic potential by inhibition of cellular senescence. Cell Death Dis. 8 (2), e2595. 10.1038/cddis.2017.2 28151468 PMC5386477

[B118] Tong J. Sun L. Zhu B. Fan Y. Ma X. Yu L. (2017). Pulsed electromagnetic fields promote the proliferation and differentiation of osteoblasts by reinforcing intracellular calcium transients. Bioelectromagnetics 38 (7), 541–549. 10.1002/bem.22076 28833306

[B119] Tsai M. T. Chang W. H. Chang K. Hou R. J. Wu T. W. (2007). Pulsed electromagnetic fields affect osteoblast proliferation and differentiation in bone tissue engineering. Bioelectromagnetics 28 (7), 519–528. 10.1002/bem.20336 17516509

[B120] Tschon M. Veronesi F. Contartese D. Sartori M. Martini L. Vincenzi F. (2018). Effects of pulsed electromagnetic fields and platelet rich plasma in preventing osteoclastogenesis in an *in vitro* model of osteolysis. J. Cell Physiol. 233 (3), 2645–2656. 10.1002/jcp.26143 28786478

[B121] Umiatin U. Hadisoebroto Dilogo I. Sari P. Kusuma Wijaya S. (2021). Histological analysis of bone callus in delayed union model fracture healing stimulated with pulsed electromagnetic fields (PEMF). Sci. (Cairo) 2021, 1–6. 10.1155/2021/4791172 PMC8413019 34484848

[B122] Vadlamani R. A. Nie Y. Detwiler D. A. Dhanabal A. Kraft A. M. Kuang S. (2019). Nanosecond pulsed electric field induced proliferation and differentiation of osteoblasts and myoblasts. J. R. Soc. Interface 16 (155), 20190079. 10.1098/rsif.2019.0079 31213169 PMC6597781

[B123] Valiya Kambrath A. Williams J. N. Sankar U. (2020). An improved methodology to evaluate cell and molecular signals in the reparative callus during fracture healing. J. Histochem Cytochem 68 (3), 199–208. 10.1369/0022155419900915 31928129 PMC7045301

[B124] van der Jagt O. P. van der Linden J. C. Waarsing J. H. Verhaar J. A. Weinans H. (2012). Systemic treatment with pulsed electromagnetic fields do not affect bone microarchitecture in osteoporotic rats. Int. Orthop. 36 (7), 1501–1506. 10.1007/s00264-011-1471-8 22249842 PMC3385882

[B125] Villa I. Mrak E. Rubinacci A. Ravasi F. Guidobono F. (2006). CGRP inhibits osteoprotegerin production in human osteoblast-like cells via cAMP/PKA-dependent pathway. Am. J. Physiol. Cell Physiol. 291 (3), C529–C537. 10.1152/ajpcell.00354.2005 16611736

[B126] Wang P. Liu J. Yang Y. Zhai M. Shao X. Yan Z. (2017). Differential intensity-dependent effects of pulsed electromagnetic fields on RANKL-induced osteoclast formation, apoptosis, and bone resorbing ability in RAW264.7 cells. Bioelectromagnetics 38 (8), 602–612. 10.1002/bem.22070 28741320

[B127] Wang W. Yeung K. W. K. (2017). Bone grafts and biomaterials substitutes for bone defect repair: a review. Bioact. Mater 2 (4), 224–247. 10.1016/j.bioactmat.2017.05.007 29744432 PMC5935655

[B128] Wang Y. Nishida S. Elalieh H. Z. Long R. K. Halloran B. P. Bikle D. D. (2006). Role of IGF-I signaling in regulating osteoclastogenesis. J. Bone Min. Res. 21 (9), 1350–1358. 10.1359/jbmr.060610 PMC10723110 16939393

[B129] Wang Y. Y. Pu X. Y. Shi W. G. Fang Q. Q. Chen X. R. Xi H. R. (2019). Pulsed electromagnetic fields promote bone formation by activating the sAC-cAMP-PKA-CREB signaling pathway. J. Cell Physiol. 234 (3), 2807–2821. 10.1002/jcp.27098 30067871

[B130] Wei Y. Xiaolin H. Tao S. (2008). Effects of extremely low-frequency-pulsed electromagnetic field on different-derived osteoblast-like cells. Electromagn. Biol. Med. 27 (3), 298–311. 10.1080/15368370802289604 18821205

[B131] Wiesmann H. Hartig M. Stratmann U. Meyer U. Joos U. (2001). Electrical stimulation influences mineral formation of osteoblast-like cells *in vitro* . Biochim. Biophys. Acta 1538 (1), 28–37. 10.1016/s0167-4889(00)00135-x 11341980

[B132] Xie Y. F. Shi W. G. Zhou J. Gao Y. H. Li S. F. Fang Q. Q. (2016). Pulsed electromagnetic fields stimulate osteogenic differentiation and maturation of osteoblasts by upregulating the expression of BMPRII localized at the base of primary cilium. Bone 93, 22–32. 10.1016/j.bone.2016.09.008 27622883

[B133] Xu J. Wang J. Chen X. Li Y. Mi J. Qin L. (2020). The effects of calcitonin gene-related peptide on bone homeostasis and regeneration. Curr. Osteoporos. Rep. 18 (6), 621–632. 10.1007/s11914-020-00624-0 33030684

[B134] Xu J. Wang W. Clark C. C. Brighton C. T. (2009). Signal transduction in electrically stimulated articular chondrocytes involves translocation of extracellular calcium through voltage-gated channels. Osteoarthr. Cartil. 17 (3), 397–405. 10.1016/j.joca.2008.07.001 18993082

[B135] Yan Z. Wang D. Cai J. Shen L. Jiang M. Liu X. (2022). High-specificity protection against radiation-induced bone loss by a pulsed electromagnetic field. Sci. Adv. 8 (34), eabq0222. 10.1126/sciadv.abq0222 36001662 PMC9401628

[B136] Yang J. Zhang J. Ding C. Dong D. Shang P. (2018). Regulation of osteoblast differentiation and iron content in mc3t3-E1 cells by static magnetic field with different intensities. Biol. Trace Elem. Res. 184 (1), 214–225. 10.1007/s12011-017-1161-5 29052173 PMC5992240

[B137] Yang J. C. Lee S. Y. Chen C. A. Lin C. T. Chen C. C. Huang H. M. (2010). The role of the calmodulin-dependent pathway in static magnetic field-induced mechanotransduction. Bioelectromagnetics 31 (4), 255–261. 10.1002/bem.20559 19953573

[B138] Yasuda I. (1977a). The classic: fundamental aspects of fracture treatment by Iwao Yasuda, reprinted from J. Kyoto Med. Soc., 4:395-406, 1953. Clin. Orthop. Relat. Res. 124, 5–8. 10.1097/00003086-197705000-00002 340088

[B139] Yasuda I. (1977b). Electrical callus and callus formation by electret. Clin. Orthop. Relat. Res. 124, 53–56. 10.1097/00003086-197705000-00007 598093

[B140] Yonemori K. Matsunaga S. Ishidou Y. Maeda S. Yoshida H. (1996). Early effects of electrical stimulation on osteogenesis. Bone 19 (2), 173–180. 10.1016/8756-3282(96)00169-x 8853862

[B141] Yu Y. Wang Y. Zhang W. Wang H. Li J. Pan L. (2020). Biomimetic periosteum-bone substitute composed of preosteoblast-derived matrix and hydrogel for large segmental bone defect repair. Acta Biomater. 113, 317–327. 10.1016/j.actbio.2020.06.030 32574859

[B142] Yuan J. Xin F. Jiang W. (2018). Underlying signaling pathways and therapeutic applications of pulsed electromagnetic fields in bone repair. Cell Physiol. Biochem. 46 (4), 1581–1594. 10.1159/000489206 29694967

[B143] Yuen-Chi Lau R. Qian X. Po K. T. Li L. M. Guo X. (2017). Response of rat tibia to prolonged unloading under the influence of electrical stimulation at the dorsal root ganglion. Neuromodulation 20 (3), 284–289. 10.1111/ner.12488 27578548

[B144] Yumoto H. Hirao K. Tominaga T. Bando N. Takahashi K. Matsuo T. (2015). Electromagnetic wave irradiation promotes osteoblastic cell proliferation and up-regulates growth factors via activation of the ERK1/2 and p38 MAPK pathways. Cell Physiol. Biochem. 35 (2), 601–615. 10.1159/000369722 25612851

[B145] Zhai M. Jing D. Tong S. Wu Y. Wang P. Zeng Z. (2016). Pulsed electromagnetic fields promote *in vitro* osteoblastogenesis through a Wnt/β-catenin signaling-associated mechanism. Bioelectromagnetics 37 (3), 152–162. 10.1002/bem.21961 26891468

[B146] Zhang J. Xu H. Han Z. Chen P. Yu Q. Lei Y. (2017). Pulsed electromagnetic field inhibits RANKL-dependent osteoclastic differentiation in RAW264.7 cells through the Ca(2+)-calcineurin-NFATc1 signaling pathway. Biochem. Biophys. Res. Commun. 482 (2), 289–295. 10.1016/j.bbrc.2016.11.056 27856256

[B147] Zhang M. Matinlinna J. P. Tsoi J. K. H. Liu W. Cui X. Lu W. W. (2020). Recent developments in biomaterials for long-bone segmental defect reconstruction: a narrative overview. J. Orthop. Transl. 22, 26–33. 10.1016/j.jot.2019.09.005 PMC7231954 32440496

[B148] Zhang X. Liu X. Pan L. Lee I. (2010). Magnetic fields at extremely low-frequency (50 Hz, 0.8 mT) can induce the uptake of intracellular calcium levels in osteoblasts. Biochem. Biophys. Res. Commun. 396 (3), 662–666. 10.1016/j.bbrc.2010.04.154 20438704

[B149] Zhang X. Zhang C. Lin Y. Hu P. Shen Y. Wang K. (2016). Nanocomposite membranes enhance bone regeneration through restoring physiological electric microenvironment. ACS Nano 10 (8), 7279–7286. 10.1021/acsnano.6b02247 27389708

[B150] Zhao M. Song B. Pu J. Wada T. Reid B. Tai G. (2006). Electrical signals control wound healing through phosphatidylinositol-3-OH kinase-gamma and PTEN. Nature 442 (7101), 457–460. 10.1038/nature04925 16871217

[B151] Zhong C. Zhao T. F. Xu Z. J. He R. X. (2012). Effects of electromagnetic fields on bone regeneration in experimental and clinical studies: a review of the literature. Chin. Med. J. Engl. 125 (2), 367–372. 22340573

[B152] Zhou J. Gao Y. H. Zhu B. Y. Shao J. L. Ma H. P. Xian C. J. (2019). Sinusoidal electromagnetic fields increase peak bone mass in rats by activating wnt10b/β‐catenin in primary cilia of osteoblasts. J. Bone Min. Res. 34 (7), 1336–1351. 10.1002/jbmr.3704 30779853

[B153] Zhou J. Li X. Liao Y. Feng W. Fu C. Guo X. (2015). Pulsed electromagnetic fields inhibit bone loss in streptozotocin-induced diabetic rats. Endocrine 49 (1), 258–266. 10.1007/s12020-014-0439-z 25273319

[B154] Zhou J. Ma X. N. Gao Y. H. Yan J. L. Shi W. G. Xian C. J. (2016). Sinusoidal electromagnetic fields promote bone formation and inhibit bone resorption in rat femoral tissues *in vitro* . Electromagn. Biol. Med. 35 (1), 75–83. 10.3109/15368378.2014.971958 25333898

[B155] Zhou J. Ming L. G. Ge B. F. Wang J. Q. Zhu R. Q. Wei Z. (2011). Effects of 50 Hz sinusoidal electromagnetic fields of different intensities on proliferation, differentiation and mineralization potentials of rat osteoblasts. Bone 49 (4), 753–761. 10.1016/j.bone.2011.06.026 21726678

[B156] Zhou J. Wang J. Q. Ge B. F. Ma X. N. Ma H. P. Xian C. J. (2014). Different electromagnetic field waveforms have different effects on proliferation, differentiation and mineralization of osteoblasts *in vitro* . Bioelectromagnetics 35 (1), 30–38. 10.1002/bem.21794 23775573

[B157] Zhu Y. Goh C. Shrestha A. (2021). Biomaterial properties modulating bone regeneration. Macromol. Biosci. 21 (4), e2000365. 10.1002/mabi.202000365 33615702

